# Alectinib Monotherapy in Isolated Central Nervous System Relapse of ALK-Positive Anaplastic Large Cell Lymphoma

**DOI:** 10.1155/2022/4749452

**Published:** 2022-02-03

**Authors:** Julian Verran, Vidya Mathavan

**Affiliations:** Department of Haematology, Waikato Hospital, 183 Pembroke Street, Hamilton 3204, New Zealand

## Abstract

Anaplastic lymphoma kinase-positive anaplastic large cell lymphoma (ALK-positive ALCL) is an aggressive form of peripheral T cell lymphoma (PTCL), harbouring an underlying pathogenic ALK fusion gene that produces a constitutively activated tyrosine kinase. The ALK tyrosine kinase provides a targeted treatment option in the form of ALK inhibitors. ALK-positive ALCL may rarely involve the central nervous system (CNS), either at presentation or on relapse of disease. The outcomes of CNS relapse in PTCL are historically extremely poor. We present a case of a 20-year-old man diagnosed with stage IVB ALK-positive ALCL. He responded favourably to six cycles of cyclophosphamide, doxorubicin, vincristine, etoposide, and prednisone (CHOEP). He unfortunately relapsed with isolated CNS involvement only 3 weeks after completing his sixth cycle of CHOEP chemotherapy. The CNS-penetrating ALK inhibitor alectinib was identified as a targeted treatment option, as he was not a candidate for CNS-penetrating chemotherapy due to significant iatrogenic renal impairment. After commencing alectinib monotherapy, he rapidly achieved a clinical and radiological response. He has now remained in a durable remission for more than two years on alectinib monotherapy.

## 1. Introduction

ALCL is an aggressive form of PTCL. It is characterised morphologically by the presence of large anaplastic cells that express CD30. ALCL can be categorised into two distinct entities based on the presence or absence of a rearrangement in the ALK gene. The majority of cases of ALCL (up to 80%) harbour a rearrangement in the ALK gene located on chromosome 2p23 [1] that leads to the formation of a pathogenic fusion gene that produces a constitutively activated tyrosine kinase, promoting cell growth and inhibiting apoptosis [1, 2]. The most common ALK translocation partner is the nucleophosmin (NPM) gene on chromosome 5q35, forming the NPM-ALK fusion gene [1]; other ALK translocation partners have also been identified. The presence of an ALK rearrangement in ALCL infers a better prognosis compared with ALK-negative ALCL.

ALK-positive ALCL can rarely relapse in the CNS, and this is historically associated with survival measured in short months. There is no standard treatment approach to CNS relapse of ALK-positive ALCL; however, the development of targeted treatment in the form of CNS-penetrating ALK inhibitors provides some hope. Alectinib is a CNS-penetrating, second-generation, oral ALK tyrosine kinase inhibitor used successfully to treat ALK-positive non-small cell lung cancer with CNS involvement [3]. To our knowledge, there have only been two published case reports of the use of alectinib monotherapy in patients with CNS involvement of ALK-positive ALCL [4, 5].

## 2. Case Presentation

A 20-year-old male presented to the emergency department with a one-month history of recurrent fevers, night sweats, pleuritic chest pain, and progressive multifocal lymphadenopathy. Routine blood tests revealed leuconeutrophilia, cholestatic derangement of liver enzymes, and elevated lactate dehydrogenase and C-reactive protein. He was commenced on antibiotics, and an infectious screen was performed. Chest X-ray revealed a widened superior mediastinum and prominent right hilum, suspicious for lymphadenopathy, along with a right-sided pleural effusion. Computed tomography (CT) scan confirmed mediastinal, hilar, and retroperitoneal lymphadenopathy with splenomegaly. It also demonstrated extranodal involvement in the form of pulmonary nodules and pleural enhancement with an associated right-sided effusion. A pleural fluid aspirate was obtained, along with a fine needle aspirate via endobronchial ultrasound; however, both were inconclusive. An open biopsy of a right neck nodal mass was then performed under general anaesthesia.

The histology showed sheets of intermediate-sized abnormal lymphoid cells with abundant cytoplasm and irregular nuclear membranes. The abnormal lymphocytes expressed CD30, BCL2, and EMA by immunohistochemistry (IHC) and had patchy expression of CD3. The cells also had strong nuclear expression of ALKp80 by IHC. The cells did not express CD10 or CD20. The morphological and immune-histochemical features of the biopsy were consistent with ALK-positive ALCL. Dedicated fluorescence in situ hybridisation studies for ALK gene rearrangement was not performed due to the strong IHC positivity. Additional workup included a bone marrow biopsy that was negative for lymphoma involvement. An upfront lumbar puncture was not performed. The patient was diagnosed with stage IVB ALK-positive ALCL, and the disease was classified as high-risk based on his international prognostic index score of 4 (>1 extranodal site, advanced stage, elevated LDH, and performance score on admission of 2).

The patient was commenced on high-dose prephase prednisone 80 mg daily for 3 days followed by CHOEP-14 systemic chemotherapy (cyclophosphamide 750 mg/m^2^, doxorubicin 50 mg/m^2^, vincristine 1.4 mg/m^2^ (capped at 2 mg), etoposide 100 mg/m^2^ on days 1–3 (days 2-3 given orally), and prednisone 100 mg/day days 1–5, with PEG-filgrastim support). He was planned for six cycles of CHOEP-14 and was not planned for upfront CNS prophylaxis.

The patient had a rapid clinical improvement on systemic chemotherapy. An interim CT scan performed after 3 cycles of chemotherapy confirmed a complete response with resolution of his lymphadenopathy and splenomegaly.

**Figure 1:**
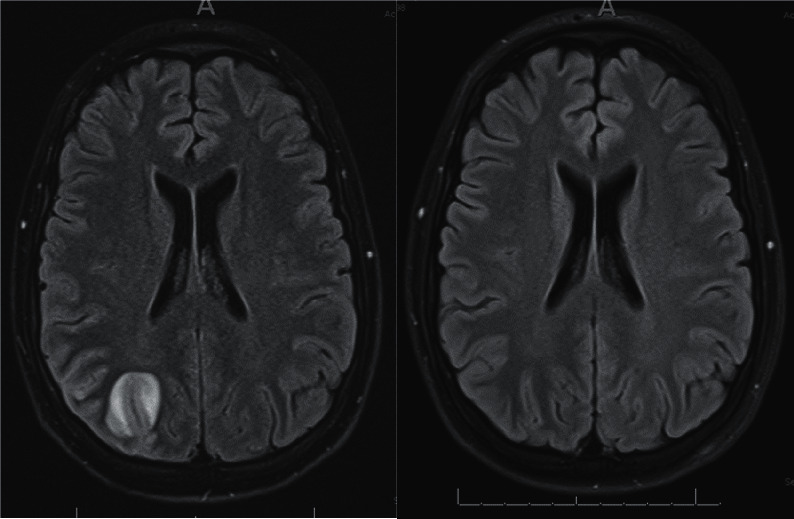
MRI brain at time of relapse (August 2019), on the left, demonstrating a 3.4 cm enhancing lesion within the parietal region with associated leptomeningeal enhancement. On the right, a repeat MRI brain (January 2020) after 3 months on alectinib monotherapy showing resolution of lesion and leptomeningeal enhancement.

**Figure 2:**
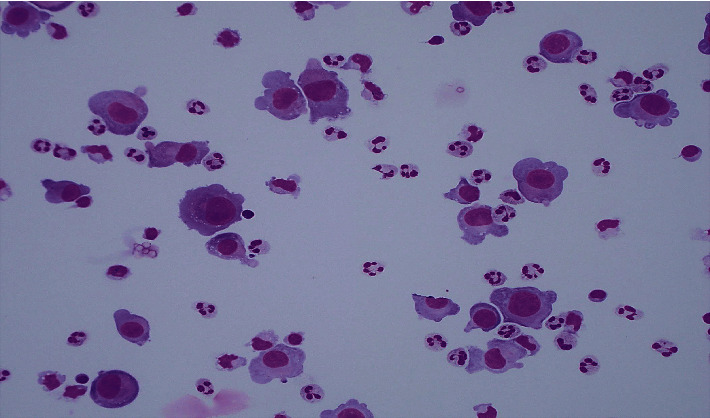
Cerebrospinal fluid analysis at time of isolated CNS relapse in August 2019 showing diffuse involvement by lymphoma cells.

Three weeks after completing his 6^th^ cycle of CHOEP systemic chemotherapy, the patient represented acutely to the emergency department with a 2 week history of fevers and severe headaches that were associated with vomiting and photophobia. An urgent CT brain with contrast revealed mild right parietal leptomeningeal enhancement. The CT scan was followed by dedicated magnetic resonance imaging (MRI) of his brain. The MRI demonstrated an abnormal 3.4 cm lesion with high T2 signal change in the right parietal cortex, along with associated leptomeningeal enhancement (Figure 1). A lumbar puncture was performed, and the CSF demonstrated large numbers of abnormal lymphocytes (Figure 2) that expressed CD30 by flow cytometry. They also had variable expression of CD7, coexpressed CD4/8, had reduced expression of CD3, and were negative for CD5. Dexamethasone was commenced. A CT of his neck, chest, abdomen, and pelvis excluded systemic relapse of disease, and the patient was confirmed as having an isolated CNS relapse of his ALK-positive ALCL.

The patient was commenced on salvage chemotherapy two days later as per the protocol developed by Korfel et al. [6] for CNS relapse of aggressive lymphomas (methotrexate 4 g/m^2^ intravenously (IV) day 1, ifosfamide 2 g/m^2^ IV days 3–5, and oral dexamethasone 4 mg bd on days 6–10. This induction course is repeated on day 22 in those patients without clinical progression) with a plan to proceed to autologous stem cell transplant. The salvage chemotherapy was to be administered along with concurrent twice weekly intrathecal (IT) chemotherapy (alternating IT methotrexate 12.5 mg and IT cytarabine 50 mg) for 2 weeks.

Two days into his salvage treatment, the patient developed an iatrogenic acute kidney injury following the administration of high-dose IV methotrexate, delaying the ifosfamide by several days. After review of the clinical situation, a decision was made to suspend the salvage chemotherapy and trial compassionate access alectinib (600 mg bd), a second-generation ALK inhibitor with good CNS penetration. Importantly, at the time of commencing alectinib monotherapy, the patient's CSF was still positive for disease by flow cytometry.

The patient's symptoms quickly resolved on the alectinib monotherapy, and multiple further CSF analyses were clear of lymphoma cells. A follow-up MRI brain 3 months after commencing alectinib confirmed that the patient was in complete radiological remission (Figure 1). The patient has had no recognised adverse effects to alectinib.

At a routine outpatient clinic appointment 12 months after commencing alectinib, the patient complained of intermittent headaches for the preceding 3 weeks. A repeat MRI brain and CSF analysis confirmed ongoing remission. The patient remains well on alectinib monotherapy, more than two years following his CNS relapse.

## 3. Discussion

We present a rare case of isolated CNS relapse of ALK-positive ALCL in a patient who had initially responded well to CHOEP systemic chemotherapy. CNS involvement of PTCL and ALK-positive ALCL is rare, with estimates between 2% and 6% [7, 8]. A Swedish population-based study found that 4.5% of patients (28/625) with PTCL had CNS relapse of disease [8]. Of the patients in the study with ALK-positive ALCL, 6% (3/48) experienced CNS relapse.

The outcomes of CNS relapse in PTCL are historically extremely poor, with survival estimates between 1 and 7 months [7]. The only risk factor for CNS involvement consistently identified in the limited studies available is ≥2 extranodal sites of disease [7–9]. ALK-positive ALCL with ≥2 extranodal sites of disease is considered high-risk for early CNS relapse [7]. Chihara et al. performed a retrospective analysis of CNS risk by PTCL subtype in 616 patients. They identified 18 patients that had both ALK-positive ALCL and ≥2 extranodal sites of disease. 4 of these 18 patients had CNS relapse, giving a one-year CNS relapse risk of 15% [7]. This in hindsight suggests that our patient should have had upfront CNS analysis and CNS prophylaxis therapy due to the lung and pleural involvement.

When CNS relapse does occur in ALK-positive ALCL, there is no standard salvage approach. Given this lack of standard approach, we had initially planned to use a CNS-penetrating chemotherapy protocol developed by Korfel et al. [6], followed by a consolidative autologous stem cell transplant (ASCT). This protocol has shown reasonable results in treating CNS relapse of aggressive lymphomas with a 2-year OS of 63% for all patients and 68% for those completing an ASCT [6]. Of the patients included in the study, 10% (3/30) were PTCLs. Unfortunately, the development of an iatrogenic kidney injury in our patient precluded us completing the protocol.

Two potential targets for novel treatment in ALK-positive ALCL is the CD30 antigen and ALK tyrosine kinase. Brentuximab vedotin is used in relapsed/refractory systemic ALCL; however, there is no evidence to suggest it can penetrate the blood-brain barrier [10]. The constitutively activated ALK tyrosine kinase in ALK-positive ALCL was a logical alternative target, using the second-generation, CNS-penetrating, ALK tyrosine kinase inhibitor alectinib. Alectinib has demonstrated good efficacy in patients with CNS involvement of ALK-positive non-small cell lung cancer by averting the progression of baseline CNS metastases and preventing the development of new CNS lesions [3]. We were able to obtain alectinib on a compassionate access scheme for our patient.

To our knowledge, this is the third case report of a patient treated with alectinib monotherapy for CNS involvement of ALK-positive ALCL. In the first case, Reed et al. [5] presented a patient who had CNS involvement at presentation and was treated with CHOEP systemic chemotherapy and high-dose methotrexate (HD-MTX) for CNS involvement. The patient experienced disease progression whilst on this regimen and was subsequently commenced on alectinib. The patient completed 6 months of alectinib monotherapy, with imaging and CSF analysis showing complete response. The patient proceeded to a matched unrelated donor allogeneic stem cell transplant (allo-SCT), with the alectinib being discontinued at day of conditioning. The patient quickly relapsed on day 30 post-transplant. The patient was recommenced on alectinib with a quick clinical response.

In the second case, Tomlinson et al. [4] presented a patient that relapsed with cerebellar involvement of ALK-positive ALCL soon after completing systemic treatment with CHOEP chemotherapy. The patient completed 2 cycles of HD-MTX followed by alectinib monotherapy. Follow-up MRI confirmed radiological remission. The patient was planned for an allo-SCT and, however, had infectious complications that precluded this. At the time of publishing, the patient had endured >12 months of remission on alectinib monotherapy.

In summary, our patient with isolated CNS involvement of ALK-positive ALCL has to date (>24 months) been successfully treated with alectinib monotherapy. This remission has been achieved without concurrent systemic chemotherapy or consolidative stem cell transplantation. Alectinib appears to be an efficacious and well tolerated treatment option, in the setting of isolated CNS relapse of ALK-positive ALCL. There is no evidence base to guide us on long-term probabilities of relapse or cure on alectinib for our patient. We therefore do not know whether his durable remission should now be consolidated with an autologous or allogeneic stem cell transplant. We also do not know whether alectinib can be safely discontinued at any point in the future.

## Data Availability

No data were used to support this case report.

## References

[1] Hapgood G., Savage K. J. (2015). The biology and management of systemic anaplastic large cell lymphoma. *Blood*.

[2] Freedman A. S., Aster J. C. (2020). *Clinical Manifestations, Pathologic Features, and Diagnosis of Systemic Anaplastic Large Cell Lymphoma*.

[3] Nishio M., Nakagawa K., Mitsudomi T. (2018). Analysis of central nervous system efficacy in the J-ALEX study of alectinib versus crizotinib in ALK-positive non-small-cell lung cancer. *Lung Cancer*.

[4] Tomlinson S. B., Sandwell S., Chuang S. T., Johnson M. D., Vates G. E., Reagan P. M. (2019). Central nervous system relapse of systemic ALK-rearranged anaplastic large cell lymphoma treated with alectinib. *Leukemia Research*.

[5] Reed D. R., Hall R. D., Gentzler R. D., Volodin L., Douvas M. G., Portell C. A. (2019). Treatment of refractory ALK rearranged anaplastic large cell lymphoma with alectinib. *Clinical Lymphoma, Myeloma & Leukemia*.

[6] Korfel A., Elter T., Thiel E. (2013). Phase II study of central nervous system (CNS)-directed chemotherapy including high-dose chemotherapy with autologous stem cell transplantation for CNS relapse of aggressive lymphomas. *Haematologica*.

[7] Chihara D., Fanale M. A., Miranda R. N. (2018). The risk of central nervous system (CNS) relapses in patients with peripheral T-cell lymphoma. *PLoS One*.

[8] Ellin F., Landström J., Jerkeman M., Relander T. (2015). Central nervous system relapse in peripheral T-cell lymphomas: a Swedish lymphoma registry study. *Blood*.

[9] Chin C. K., Cheah C. Y. (2017). How I treat patients with aggressive lymphoma at high risk of CNS relapse. *Blood*.

[10] Kim Y., Sudo A., Oyama R. (2020). Isolated central nervous system progression during systemic treatment with brentuximab vedotin monotherapy in a pediatric patient with recurrent ALK-negative anaplastic large cell lymphoma. *Journal of Pediatric Hematology*.

